# Ethical considerations regarding penis transplantation surgery in transgender men—an empirical ethics study

**DOI:** 10.1093/sexmed/qfad041

**Published:** 2023-09-14

**Authors:** Kristin B de Haseth, Anne M Gehrels, Guy Widdershoven, Mark-Bram Bouman, Tim C van de Grift

**Affiliations:** Department of Plastic, Gender, Reconstructive and Hand Surgery, Amsterdam UMC Location VUmc, Amsterdam, The Netherlands; Center of Expertise on Gender Dysphoria, Amsterdam UMC, Location VUmc, Amsterdam, The Netherlands; Amsterdam Public Health Institute, Amsterdam UMC Location VUmc, Amsterdam, The Netherlands; Department of Plastic, Gender, Reconstructive and Hand Surgery, Amsterdam UMC Location VUmc, Amsterdam, The Netherlands; Department of Ethics, Law and Humanities, Amsterdam UMC, VU University, Amsterdam, The Netherlands; Amsterdam Public Health Institute, Amsterdam UMC Location VUmc, Amsterdam, The Netherlands; Department of Ethics, Law and Humanities, Amsterdam UMC, VU University, Amsterdam, The Netherlands; Department of Plastic, Gender, Reconstructive and Hand Surgery, Amsterdam UMC Location VUmc, Amsterdam, The Netherlands; Center of Expertise on Gender Dysphoria, Amsterdam UMC, Location VUmc, Amsterdam, The Netherlands; Amsterdam Public Health Institute, Amsterdam UMC Location VUmc, Amsterdam, The Netherlands; Department of Plastic, Gender, Reconstructive and Hand Surgery, Amsterdam UMC Location VUmc, Amsterdam, The Netherlands; Center of Expertise on Gender Dysphoria, Amsterdam UMC, Location VUmc, Amsterdam, The Netherlands; Amsterdam Public Health Institute, Amsterdam UMC Location VUmc, Amsterdam, The Netherlands

**Keywords:** ethics study, transgender men, penis transplantation surgery, gender dysphoria, gender reassignment surgery, penis transplantation

## Abstract

**Background:**

The ongoing quest to surgically create the (nearly) ideal neophallus in transgender men has led to the continuous development of transgender medicine and the proposed introduction of penis transplantation. However, both technical and ethical issues arise when developing this treatment.

**Aim:**

We sought to extract ethical considerations among different stakeholder groups regarding penile transplantation surgery in transgender men and to define preliminary clinical recommendations.

**Methods:**

Three semistructured focus groups, consisting of different stakeholders, were organized to participate in discussions of ethical considerations retrieved from the ethics literature of transplantation and transgender medicine. Analysis of the results was performed according to empirical ethics.

**Outcomes:**

Study outcomes were the identification of qualitative themes describing ethical considerations pertaining to penile transplantation in transgender men.

**Results:**

Nineteen people participated in this qualitative study. The main domains that emerged included arguments in favor of and arguments against penile transplantation. Although the participants expressed positive attitudes toward developing this care, with acknowledgement of the current limitations stakeholders of all groups were reluctant to endorse the introduction of penile transplantation for transgender men at this point.

**Clinical Implications:**

Under the present circumstances, all groups expressed both a wide variety of ethical considerations as well as a tendency to prefer developing alternative treatment options or improving the results of currently available treatments in relation to penile transplantation for transgender men.

**Strengths and Limitations:**

This study was the first (empirical) study to focus on this topic and included a diversity of stakeholder perspectives. Limitations included the hypothetical nature of the discussion as well as the clinical setting in which the topic has been studied.

**Conclusion:**

Introducing penile transplantation for transgender men, under the current circumstances, comes with a wide range of ethical considerations, which deal with personal identity, autonomy, medical risks, risk for transgender support and donor willingness, and issues regarding equality. Despite the present hesitancy to use penile transplantation, should the technical side of this treatment option develop, further research in the ethical field of penile transplantation for transgender men is recommended.

## Introduction

Gender dysphoria is characterized by a marked discrepancy between birth-assigned sex and gender identity and is associated with bodily and emotional distress.^1^ As a result, a subgroup of individuals experiencing gender dysphoria might seek gender-affirming medical care, which may include genital gender-affirming surgeries to align gender identity and bodily characteristics.^2^ The wish to be a “complete” man, including male-typical genital appearance and function, is generally one of the important motivators for transgender men to opt for the current gold standard genital gender-affirming surgery, the phalloplasty. The aim of the phalloplasty is to create a phallus that resembles the biological penis while retaining tactile and erogenous sensation, ensuring rigidity for penetrative sex, and possibly enabling standing voiding, while avoiding complications such as low donor site comorbidity or stigmatizing scars. Studies have shown that transgender men generally report a good quality of life after gender-affirming surgery.^3^^,^^4^ Nonetheless, the phalloplasty procedure has multiple drawbacks, including a high complication rate, voiding issues, cosmetic imperfections, and disappointing penetrative ability, which may explain why fewer than a third of transgender men opt for this treatment modality.^4–6^ As part of continuous optimization of surgical care for this group, there is a need to explore new surgical options for penile reconstruction in transgender men.

For some years, transplantation medicine has been considered one of the possible directions of a new treatment paradigm in transgender medicine.^7^ Partially, this is the result of substantial changes in the field of human transplantation: vascular composite allografts (VCAs) are supplementing traditional life-saving solid organ transplants.^8^ A new form of transplantation, VCA involves transplantation of non–life saving organs such as facial tissue and hands. Forty (partial) face transplants have been performed and more than 100 individuals have received hand transplants worldwide.^8–11^ Children have been born to women who received a transplanted uterus from their mother or sister.^8^^,^^12^ Along with these developments, there has been a shift in thinking with regard to indications for transplantations, which increasingly include motivations that are not solely related to lifesaving but may also include improvement of a person’s quality of life.^8^

Another VCA procedure being developed is penis transplantation. Worldwide, five penis transplants have been (successfully) performed in cisgender men.^13–18^ The first human penile transplant was attempted in China in 2006. Although a technical success, the graft was explanted after 14 days due to psychological rejection.^13^^,^^14^ Following this first case, scholars criticized the patient selection process and surgical technique used with this approach.^19^ However, penile transplantation is still believed to potentially offer the best aesthetic and functional outcomes in penile reconstruction^19^ and is being further developed in South Africa and the United States.^15^

The aforementioned developments in cisgender men have led to the exploration of applying penis transplantation in transgender men as well, although this procedure is in the early stages of development. Selvaggi et al. have shown that it is possible to explant the penis and associated vessels, nerves, and urethra en bloc from a cadaver.^20^ These authors concluded that this technique would enable penile transplantation from a cadaveric donor male to a recipient with natal female pelvic anatomy, a finding that has been previously confirmed by Tiftikcioglu et al.^21^

Despite the surgical potential of penile transplantation in transgender men, more experimental, immunologic, psychological, and also ethical research is needed before attempting this novel technique. Explorative studies on the ethical considerations regarding other VCA procedures indicated that overall concepts such as personal identity, autonomy, and risk and benefit ratio need to be considered when introducing such care.^8^^,^^22–24^ Scholars mention that weighing of risks and benefits and anticipating social impact are among the most important ethical considerations.^23^^,^^25^ To define the possible position of penis transplantation within transgender care and healthcare at large, ethical research can identify what principles different stakeholders see as relevant and how to weigh possible risks and benefits and help to define guidelines on indications and prioritization of candidates.^26^^,^^27^

As this information is critical to further developing this care but is currently lacking, our aim in the present study was to investigate the ethical considerations among different stakeholder groups regarding penile transplantation surgery in transgender men and define preliminary clinical recommendations for further treatment development. Is penile transplantation, from an ethical point of view, a viable treatment option for transgender men desiring to have male genitalia?

## Materials and methods

### Research team

A qualitative study using semi-structured focus groups was conducted in the Amsterdam University Medical Center (location VUmc) between June 2018 and February 2020. This study was designed by the department of Plastic, Reconstructive, and Hand Surgery, in collaboration with the Bioethics department and approved by the Institutional Review Board of the Amsterdam University Medical Center (location VUmc). The research team included a plastic surgeon and PhD candidate, a clinical intern with a master’s degree in bioethics, and a postdoctoral researcher and physician in psychiatry, all working in transgender care. There was no conflict of interest between the participants and the researchers or the researchers and the studied topic. The research was performed according to the COREQ (consolidated criteria for reporting qualitative research) guidelines.

### Study design

#### Theoretical framework

The study was set up according to the theory of empirical ethics, in which practice is used as a source of ethics, and experiences in practice can inform the process of developing normative clinical guidelines.^28^ A literature search was carried out in the fields of transgender care ethics and transplantation ethics to extract themes to be explored during the focus groups. A broad selection was made due to the explorative character of the study. The following themes were extracted and used as guidelines for the interviews during the focus groups: benefits and risks, autonomy (the ability to make one’s own decisions) and personal identity (the concept one develops about oneself that evolves over the course of one’s life), and bodily integrity (one’s right to autonomy and self-control over one’s body).^22^^,^^29^^,^^30^

#### Participant selection and setting

Data were collected though focus groups. Hence it was possible to perceive different perspectives on the subject while at the same time investigating and exploring the underlying ideas and motives. Three different stakeholder groups were identified in advance to participate in the focus groups: transgender men in different phases of medical transitioning (T), healthcare providers with experience in transgender and/or in transplantation medicine (H), and cisgender men without specific knowledge on transgender medicine who were either active donors or not (C) (Table 1). A total of 20 transgender men, all receiving care in the Amsterdam University Medical Center (location VUmc), were approached by telephone, of whom 6 were willing to participate. Also, 16 healthcare providers, all employed by the Amsterdam University Medical Center (location VUmc), the transplantation department or Center of Expertise on Gender Dysphoria, were approached by email (by A1 and A2), of whom 6 consented to participate. Last, 9 cisgender men were approached by telephone or email (by A1, A2, or A5) of whom 7 were able to participate. All eligible participants had to be at least 18 years old, sufficient in the Dutch language, and able to understand the study and provide informed consent.

**Table 1:** Sample characteristics.

Participant ID	Profession	Remarks
Transgender men		Surgeries undergone
T1	Website designer	Mastectomy, hysterectomy, gonadectomy
T2	Artist	Mastectomy, hysterectomy, gonadectomy, vaginectomy, metoidioplasty, phalloplasty
T3	Nurse in district team	Mastectomy, hysterectomy, gonadectomy, vaginectomy, phalloplasty, testis prothesis
T4	IT-consultant	Mastectomy
T5	X-ray lab technician	Mastectomy, gonadectomy, vaginectomy, phalloplasty, testis implantation
T6	Trauma nurse at defense	Mastectomy, hysterectomy, tubectomy, vaginectomy
Healthcare providers		Field of expertise
H1	Endocrinologist	Transgender care
H2	Psychologist	Transgender care
H3	Psychologist	Transgender care
H4	Nephrologist	Transplant medicine
H5	Vascular surgeon	Transplant medicine
H6	Plastic surgeon	Transgender care
Cisgender men		Donor status
C1	Business student	Active donor
C2	Sales manager	Uncertain
C3	Police officer	Active donor
C4	Doctor and researcher	Active donor
C5	Business intern	Uncertain
C6	Physics student	Not a donor
C7	Entrepreneur	Active donor

Abbreviations: C, cisgender men without specific knowledge oof transgender medicine who were either active donors or not; H, healthcare providers with experience in transgender and/or in transplantation medicine; T, transgender men in different phases of medical transitioning.

#### Procedure

Upon obtaining written informed consent from the participants, the researchers (A1, A2, and A5) scheduled a focus group, at a time when at least 6 participants were available. Three focus groups were conducted (one for each target group, see Appendix 1) and chaired by two of the first, second, or last authors (A1, A2, and A5). The focus groups were held in X and were all 180 minutes in length. All focus groups were conducted at the hosting hospital. Participants received a free meal and travel compensation only. The healthcare providers and the transgender men focus groups were held in the same period, before analysis of the data, whereas the cisgender men focus group was held after interim analysis of the data abstracted from the first two focus groups. The latter group was mainly asked about donorship and social perspective in contrast to the other groups, who were direct stakeholders in healthcare or involved as transmen. During the focus group, one interviewer took field notes and small notes were made by the participants. The focus groups were audio-recorded, transcribed verbatim by one author (A2) and coded by two authors (A2 and A5). References to names were anonymized and the recordings were erased afterward. Transcripts of the data were not returned to the participants for comments or correction. No repeat interviews were carried out.

#### Data collection

Data were collected through focus groups, given the noninferiority of this method compared with individual interviews and the ability to establish dialogues between different (normative) perspectives.^31^ The focus groups were held in a semi-structured fashion, starting with an introduction of the subject, then an overview of the agenda, assessment of the participants’ backgrounds, and an opening question (What do you think of the transplantation of a fully working penis as part of transgender healthcare?). Subsequently, the three predefined ethical themes extracted from the literature were discussed by asking open-ended sample questions. Prior to the focus groups, the researchers had defined these questions, which were provided during the focus group by the session chairs. Based on (non)verbal cues during all focus groups and data saturation assessed during interim analysis, the researchers estimated whether or not to introduce further topics to deepen the subjects discussed during the focus groups. Interviewers were responsible for the equal division of time per topic and participant. Data saturation was reached due to recurring topics indicated by the focus groups, and consensus was reached by the researchers. Consequently, and due to the participants’ different experiences, there was a slightly different focus on different themes during the different focus groups: during the transgender men focus group “personal identity” and “bodily integrity” were mostly discussed, during the healthcare providers focus group the “risk/benefit ratio” and “social factors” received much attention, and in the cisgender men focus group “social factors” and the “willingness to donate” were discussed most extensively. Social factors and available alternatives emerged as themes during the first focus groups and were therefore subsequently added to the protocol. In all focus groups, the session was ended with a closing question (Did you change your mind about penile transplantation in transgender healthcare? And if so, what did change your mind?). The questions were not pilot tested.

### Analysis

#### Data analysis and reporting

The interview transcripts were reviewed extensively, after which the data were coded by two authors (A2 and A5) and underwent preliminary evaluation according to explorative thematic analysis. Also, a first description of the coding tree was constructed. This preliminary coding tree was further modified as it became more informed by the addition of ethical themes extracted from the literature, until definitive domains, themes, and subthemes could be defined (Table 2) to derive meaning from the collected data. Analysis of the results was done according to empirical ethics, in which moral theory and empirical data are integrated to reach a normative conclusion. Participant quotations per theme were selected to illustrate the findings. Participants were not approached for feedback on the findings, as was collectively agreed on during the focus group. Two additional one-on-one interviews were conducted (with a physician-researcher in transplantation ethics, and with a gynecologist who specialized in transgender fertility and uterus surgery), which confirmed our data analyses. The data were processed and analyzed manually within Microsoft Word, Office 2016.

**Table 2:** Overview of domains and (sub)themes after data analysis.

Domain	Theme	Subtheme
Arguments in favor of penile transplantation	Improved personal identity of the recipient	Wholeness
		Gender affirmation
		Psychological well-being
	Autonomy and bodily integrity	Self-determination of the recipient
	Postoperative benefits	Genital functionality
		Genital aesthetics
Arguments against penile transplantation	Postoperative risks	Risks of immunosuppression
		Risk of transplant rejection
		Psychological burden of the treatments
	Risk–benefit ratio	
	Social factors	Decreased donor willingness
		Decreased transgender acceptance
		Regulatory issues
	Alternatives	

## Results

Extensive thematic analysis of the focus group data yielded two domains, “arguments in favor of penile transplantation” and “arguments against penile transplantation” (Table 2).

### Domain 1: arguments in favor of penile transplantation

#### Improved personal identity of the recipient

Several themes were brought up by the focus groups relating to the concept of *personal identity*, which deals with the philosophical questions related to being a person. In the context of transgender individuals and their medical transition, personal identity was often brought up in relation to becoming one’s self and feeling physically, emotionally, and socially affirmed in one’s experienced gender identity in contrast to one’s birth-assigned sex. Multiple participants in all focus groups emphasized the role penis transplantation could play in improving these processes. Personal identity was thought to be supported through feelings of wholeness and experiencing gender affirmation and psychological well-being, as will be discussed below.

##### Wholeness

Participants from both the healthcare provider and transgender men’s groups shared the view that a transplanted penis, being male typical and fully functioning, would improve the feeling of completeness in transgender individuals. Being whole referred to both having a physical appearance in line with the male gender identity and having complete penile functionality (ie, voiding and sexual function) associated with cisgender males. A treatment modality with these outcomes was generally viewed as the holy grail.

H1: “What I hear in many cases [as perceived by transgender men, authors] is that it [having a real penis, authors] means a lot for self-image, identity and being a complete man, ‘I have a real penis, and this contributes substantially to my self-image and self-esteem.’”

In addition, this viewpoint was confirmed in the transgender men’s group: transgender men expected a feeling of completeness if they were to receive this treatment.

T2: “The feeling when it [the transplanted penis, authors] is there. The emotional connection which makes you feel complete should be present.”

##### Gender affirmation

Another aspect of personal identity that was brought up often in favor of penis transplantation was the gender affirmative effect it was expected to have. The process of gender affirmation (being physically, psychologically, and socially recognized as the identity one experiences) is the main underpinning of medical transitioning. Participants expected that a male-typical looking and functioning penis would strongly affirm one’s identity. Gender affirmation assists people in developing a personal identity that is closest to their inner being and supports mental health. Mostly the transgender participants underlined the importance of penile surgery, and penis transplantation specifically, in addition to other medical treatments, to support gender affirmation. This additional value resulted from the special role the penis plays in masculinity and how this is a gender-affirming organ for transgender men. This also distinguished penis transplantation from transplantations of internal organs.

T5: “It also deals with the symbolism and what people think of a penis. If you look at society, a lot depends on the penis, which is also the case with a heart off course. For me it is the appearance that differentiates, and the heart is mostly a functional muscle.”

Aside from the symbolic function the penis plays in masculinity, participants also described how penile transplantation can affirm gender identity through the feeling of wholeness and by providing the penile functionality of the recipient. Both transgender men and healthcare providers expressed the concern that current phalloplasty procedures often do not fully provide this feeling of wholeness (as it was not perceived as a full-fledged alternative for a real penis) because transgender men often cannot void standing or penetrate (without another additional surgery, which also has its complications). A penis transplant was therefore expected to affirm the male gender more than the presently available phalloplasty methods.

H3: “What I often hear is that the appearance [of the phalloplasty, authors] is perceived as ugly by them [transmen, authors]. They think it does not sufficiently resemble a penis. Also, they are disappointed with not being able to get an erection by themselves and that penetration is an issue which requires additional erection prostheses.”

##### Psychological well-being

Ultimately, many transgender participants and healthcare providers believed that, risks aside, successful penis transplantation would strongly improve the psychological well-being of the recipient. There was general agreement on the emotional importance of the penis as a body part, and that this applied to both transgender and cisgender individuals.

T6: “I work in the military, and over there they surely prefer having their hand shot, rather than their penis.”

Participants expected that penis transplantation would likely improve individual psychological well-being through feeling complete and male and that the penis would reduce distress and support living in the male gender role.

H6: “Some [recipients, authors] are very happy with ‘having something in their pants’ without requiring an external prosthesis. However, if it does not look like a biological penis, some will still be uncomfortable with that.”

T5: “It is one less thing to worry about, for example, when going to the sauna or having sex with someone.”

Some cisgender participants, having been confronted with the severity of distress transgender individuals can experience, were strongly motivated toward supporting transgender medical care and/or becoming donors themselves.

C3: “I know trans individuals and have supervised students. There was a transwoman, and I have heard from her what struggle she has gone through in order to be herself and her identity. I assume it is similar in transmen. And I know how much it [her transition, authors] helped her in becoming who she is now. That is why I say I am in favor [of penis donation, authors]. I have found people who hung themselves and wrote a letter stating they could not handle it anymore. I may take a somewhat different position than others, but that has to do with my job.”

#### Autonomy and bodily integrity

A second major theme in favor of penile transplantation dealt with views on autonomy and bodily integrity. Autonomy is considered as the ability to make one’s own decisions. Bodily integrity is considered a human right and refers to regulations with regard to one’s physique. Bodily integrity is often used when discussing prohibition of unwanted interventions such as infant genital mutilation. Both values refer to the degree of self-determination. In the case of penile transplantation surgery in transgender individuals, participants mostly brought up topics related to the degree to which individuals should be able to self-determine whether or not they can receive such care.

##### Self-determination of the recipient

All participants greatly valued individual autonomy and maximal self-determination in this decision as possible. At the same time it was difficult to define generalizable measures by which this should be weighed. Underlying was the shared notion that candidates could self-determine as long as they were considered being sufficiently resilient and well-informed, and that the transplantation should be effective in solving the person’s problem.

C2: “I do not think this [penile transplantation, authors] should be some impulse, like: ‘I want a male penis,’ but instead that this individual should have gone through thorough psychological counseling and have been assisted in understanding whether this is really going to help that person.”

H2: “Everyone decides for themselves how severe their suffering is and what risks they are willing to take.”

Transgender men also speculated on a minimum age requirement but did not reach conclusive consensus on this. Considerations related to both the legal age of making certain decisions as well as to the age when one expected that candidate-recipients would be able to oversee the consequences of their decision. Participants took different stands with regard to what they prioritized.

T4: “You cannot generalize youth. I think they have to decide themselves. When you are 18, you are an adult and should be able to decide.”

T2: “But just like with driving cars; when aged under 21, they account for most accidents. Why would you be able to make such a decision at the age of 18?”

T6: “You need time to psychologically adjust. The younger you are, the more flexible you are, and you have fewer other issues in life. However, you have to have some resilience and therefore postpone your request as long as possible. […] I do not think you can make such a decision at that tender age.”

One transgender participant pointed out that such questions regarding decision-making similarly already apply to undergoing the phalloplasty, and that decisions greatly vary based on individual preferences. In addition, some participants also suggested they would involve partners and family members in their decision-making while others would prefer full self-determination.

T6: “I am currently single, but if I was in a relationship, I would still make my own decision.”

T6: “[On the issue of having children and undergoing penile transplantation, authors] That would be a different story. Then I would disadvantage other people because of my determination. […] Imagine, if you will undergo it [the transplantation, authors], you might be a father, who is ill, for your children, and in addition contract all sorts of diseases.”

#### Postoperative benefits

Along the abovementioned arguments in favor of penile transplantation, other benefits were obtaining male-typical genital function and aesthetics.

##### Genital functionality

Compared with the current gold standard, the phalloplasty, the transplanted penis was expected by both transgender men and healthcare providers to yield better functional and sexual outcomes. Voiding while standing was a desired (and gender-affirming) functionality that was generally possible after phalloplasty with urethral lengthening; however, complications were frequently experienced after urethral surgery. In that sense, being able to receive a native male urethra through transplantation was expected to be superior than the phalloplasty.

T3: “I have many urological problems [after phalloplasty, authors], mostly based on a stricture, and with that [transplantation, authors], these things would be mostly solved.”

H1: “What I hear from many transgender men is that they postpone or do not want a phalloplasty, because they believe the phalloplasty has too many limitations.”

Also, sexual function after phalloplasty was considered suboptimal by many. If successful penis transplantation would include maintaining the penile sensation and erectile function, many participants would view this as major progress in genital surgery for transgender men. This was because the current phalloplasty generally requires penile erection prosthesis, an extra surgical procedure, to enable penetration, and penile sensation is generally limited.

H2: “It is important to have a penis that can become erect, and in which the glans is the most sensitive […].”

T2: “It is all about stiffness and sensation, that is of foremost importance. It is a mechanical thing you want to use. […] I have got a good one that does not look very nice, but I feel everything that I should feel, which is more important to me.”

T3: “To me, erection is not that important. I like it that I can pee and that I can feel something.”

##### Genital aesthetics

The last theme that emerged within the domain of postoperative benefits was the male-typical appearance that penis transplantation was expected to yield for the recipient. Again, the present phalloplasty was often perceived as suboptimal in this sense, although concerns were expressed to which degree the donor penis would be congruent with the recipient and how many matching donors would be available.

T6: “I find aesthetics also very important; I do not want to walk around with a small toilet roll attached to my lower abdomen.”

T1: “In any case, there will not be a lot of young donors, so that is a problem anyways.”

### Domain 2: Arguments against penile transplantation

#### Postoperative risks

##### Risks of immunosuppression

Both transgender men and healthcare professionals agreed that the use of immunosuppressive medication and its side effects after penis transplantation bore the greatest risks for the recipient. Participants stated that the recipient has a physically healthy body that will be exposed to risks of severe medical complications.

T6: “In general you have a healthy body, whether you wished it like this or not. But you will administer medication, making it unhealthy. […] If you are 70 and only have a few years to live, I would consent, but when you are 18, I find it a shame.”

H4: “This is the disadvantage of all allogeneic transplantations. […] You would have to give quite a high and heavy dose of immunosuppressive medication which introduces complication risks, like infections and increased chance of developing malignancies.”

H5: “Imagine you transplant a penis to someone who dies in 2 years because of interstitial pneumonia resulting from immunosuppressive medication.”

Besides the complication risks of immunosuppressive medication, healthcare professionals specifically worried about the long-term therapy compliance. Decreasing strictness and motivation with regard to taking this medication were a known issue after transplant surgery, and it was unsure whether this will be the case after penile transplantation surgery (as this is a non–life-saving procedure).

H4: “What we know from transplantations […] is that carelessness with medication sneaks in. This can be the case with all patients, even in heart transplantation. Five to 10% of people with a heart transplantation lose their organ, just because they do not want to continue taking their medication after a while.”

One transgender man and one healthcare provider expressed how they thought that it was difficult to oversee the long-term consequences of choosing this intervention from the situation in which you are when deciding.

T6: “I think you should be careful with people not being well-informed and wanting to go for the ‘easy fix.’ Later they realize this is something for life. You risk tunnel vision if the penis looks nice and you want it so badly; you do not consider the negative parts.”

H2: “I think it is complicated when you want something badly or you suffer from not having it, you cannot assess well what the chance is it will not work out. You may become more focused on ‘maybe it works in my case’ or ‘we will see then, I want it now.’”

##### Risk of transplant rejection

Another physical risk that healthcare providers emphasized, while limitedly discussed by the other participants, was that regardless of adequate immunosuppressive medication, there was still a substantial possibility of transplant rejection. Such a rejection would come with severe physical (ie, possible complications, another extensive surgery) and psychological burden (ie, returning to the previous distressing situation, disappointment).

##### Psychological burden of the treatments

Although participants expected that penis transplantation could support the psychological well-being of the transplant recipient, they also anticipated the possible psychological burden of the heavy medical treatments. Transplantation was expected to require a substantial period of physical recovery and possibly dealing with complications or even transplant loss. Such an extensive process requires good psychological resilience and motivation to comply with treatment requirements.

#### Risk-benefit ratio

There was great individual variability in how the study participants weighted the individual benefits and risks of transplantation. These differences appeared to be mainly associated with the personal values and experiences of the participants rather than the participant group they belonged to. In general, participants almost unanimously leaned toward the risks side, with the immunosuppressive medication as the strongest factor.

T5: “Good that it is possible, but I would never want it. Especially because of the medication against rejection you would have to take all your life. And having something strange attached to your body, and all the risks involved. That is in fact too much.”

H2: “We still have to oversee it; at the moment, I mostly see risks and dangers. I think, generally speaking, those risks are difficult to oversee for patients, especially those risks regarding immunosuppressive medication. I also recognize this in transplantation patients. They find it difficult to oversee the whole picture as well, even in case of life-saving procedures […].”

In addition, some healthcare providers valued the risks differently given the non–life-saving nature of penis transplantation. They proposed that penis transplantation in a transgender man who receives immunosuppressive medication for other medical reasons might be ethically more justified.

H2: “The moral dilemma of immunosuppressive medication is: can you take these risks for an ‘elective’ procedure?”

H5: “Are there any transgender men with kidney failure? That would clearly be an ideal group to start with.”

#### Social factors

During the focus groups, the impact that penile transplantation could have on the societal equilibrium seemed to be among the decisive considerations.

##### Decreased donor willingness

Participants from all groups were hesitant on the number of donors who would be willing to donate their penis. The relatively small group of eligible donors (ie, younger males), the possible negative attitudes toward donating a penis, and objections to donating their penis specifically, would result in a number of donors that is possibly insufficient to supply all interested transgender men.

H4: “Yes, there will be 400 to 450 [total donors per year, authors] divided by two for males, and then excluding children. So it is going to be quite a small group to search within.”

T4: “I do not think many people will donate, and therefore supply and demand is going to be unbalanced. It is not realistic to provide this care as a hospital.”

A strong drawback that many participants expected was the possible negative effect introducing penis transplantation for transgender men could have on the perception of transplant medicine and on the willingness to donate in general. Participants were afraid that providing such care would be seen as unnecessary and unwanted, lowering the support for transplantation medicine. Also, participants were afraid that registered donors would revoke their donorship, possibly duping recipients of other (possibly life-saving) transplants.

H4: “I am specifically curious what the public opinion will be. What the effect will be on the willingness to be donor in general. Face transplants are being performed in France already, but in that case people can emphasize with the necessity; people being mutilated. This is even more in case of organ transplantation. […] One [potential donors, authors] will think they [surgeons, authors] want more donors to perform this kind of luxurious surgeries, instead of necessary ones.”

H5: “I think, if you want to make this a successful project, you have to be extremely careful with the public opinion. You do not want to lose some hundreds of kidney transplants because people say ‘forget it, I do not want to be a part of this.’”

C2: “From my perspective, I would be a donor because I think I could save lives. In case of penis transplantation, those people do have problems you may solve through transplantation; however, it does not fit into my own image of ‘I am donor because I’m going to save lives.’”

T6: “I would pass for transplantation if other people would die because of it.”

For cisgender men, although having a positive attitude toward supporting transgender care, donating to a cisgender man felt somewhat more understandable to do, and participants were more positive toward facilitating this. Truly emphasizing with the experience of transgender men was difficult for cisgender men, which made donating less likely.

C5: “In case of a biological man it is more kind of a ‘must’ and for transgender men a ‘could.’”

C6: “Since it [being transgender, authors] can be quite a leap from your own frame of reference, it can be more difficult to imagine, so you might have a different perspective.”

In line with the aforementioned transcription, cisgender men also experienced the emotional connection with the penis as a barrier toward donating. While they were aware that it was not fully rational, since they would be deceased, the idea that such an emotional organ would get a second life withheld some from donating.

C4: “[…] perhaps I find it a more awkward organ to give a second life than others. Giving your heart a second life is beautiful idea. […] I find it a bit silly, the idea, […] that ‘Willy’ will just continue living. […] And that someone can meet ‘Willy’ again.”

##### Decreased transgender acceptance

Another aspect concerned the possible effects that introducing penis transplantation surgery may have on the public opinion of transgender individuals and transgender medicine. Many participants expected a backlash of the support for transgender medicine, viewing transplantation as unnecessary and expensive, and some even expected that it may fuel overall negative attitudes toward transgender individuals in general.

T5: “If you look how society views it, there are still many negative views on transgender individuals. Even though it is unfair and factually true, I believe penis transplantation will stir up a lot. […] People with illnesses having expensive medication [which they have to pay out of their pocket, authors] will say ‘you can have yourself operated’ [and have all expenses paid for, authors]. It is unfair, but it is the public opinion.”

Healthcare professionals also described negative attitudes within the hospital, which were expected to possibly worsen in case of this type of surgery.

H5: “Even internally [in the hospital, authors], we have to beg for operating rooms [for non–transgender operations, authors], which leads to comments such as ‘we have six aneurysms on the waiting lists and all these operating rooms have to go to transgender individuals.’”

##### Regulatory issues

Regulatory questions that came up related to allocation questions, such as societal costs of penis transplantation (should we pay for this as a society?), eligibility (who should receive this care, and how to decide?), and prioritization (who should receive this care first, and how to decide?)

##### Societal costs

Recognizing the fact that this research question was studied in a country with a public universal health insurance system, which reimburses both transplantation medicine and gender-affirming care, participants raised the question whether society should pay for such a procedure for this indication. Participants mentioned that the healthcare system should be sustainable and affordable, and that penis transplantation was perhaps not the most effective way of spending public money. While others found that affordability should not be the primary argument to decide over such fundamental treatments.

T5: “We already consume quite some care, so I doubt if you would want to do so even more for the rest of your life.”

C4: “I sympathize with how much money we collectively invest to provide high-quality care. And if such a procedure and aftercare together of one individual would cost a lot of money, I doubt if this [penile transplantation, authors] should be done.”

C6: “I believe, and people are going to disagree, that it should not be publicly funded. But that would mean that only rich people would be able to do so.”

T2: “Everything that is so fundamental for your personal well-being, even medically spoken, should not be discussed financially.”

##### Eligibility

On a personal level, participants largely agreed that in principle transgender men should be able to self-select for a penis transplantation procedure. Self-determination and attaining one’s personal identity were guiding underlying principles for many. An interesting paradox emerged as well; participants believed that given the weight of this treatment it should solve a significant problem, and a recipient should experience some suffering. At the same time, and also given the weight of the treatment, participants agreed that the recipient should be in a good psychological state to cope with the process.

H2: “You want to objectify some suffering, otherwise this [penile transplantation, authors] is unnecessary, while at the same time one has to be stable enough [to undergo the transplantation, authors].”

C3: “In case other psychiatric problems will come at play, it should be stopped.”

The cisgender participants speculated on how candidates should be selected and prioritized. They agreed that a central commission should be installed to conduct gate keeping and assess whether all treatments would be performed for the right reason and on a stable person. This commission should include experts and should have some distance from daily practice.

C2: [on who should be guiding in this commission, authors] “Not surgical doctors, because surely they want a penis hanging there and they find it a nice challenge.”

##### Prioritizing

Participants commented on prioritization of one candidate-recipient over the other on the basis of two markers, candidate age and transgender vs cisgender status. Some mentioned that younger candidates should be prioritized over older ones, while others stated that candidates whose characteristics matched the donor (ie, age and ethnicity) should be prioritized. Both transgender and cisgender participants agreed that cisgender candidates should be prioritized over transgender candidates, as they assumed higher suffering because they once had a penis and now lost it.

T2: “If you already had it [a penis, authors] I think you should be prioritized. Psychologically he has had it for whole his life. […] The whole physical built is there and perhaps it is really traumatizing for him not to have it.”

Again, the cisgender participants believed that a commission should determine systematically and objectively who to prioritize, although indicators by which this should happen were difficult to define.

C1: “There has to be some kind of card system with a matrix in which you will receive a final sum score which determines your position on the list. If you will vote it will be based on opinions […].”

#### Alternatives

The last argument against transplantation that emerged from all three focus groups was the notion whether this treatment modality should be developed over others. In the context of gender-affirming care, participants questioned what the net added value of penis transplantation would be compared with the current gold standard, phalloplasty, and if the scarce resources were most effectively spent when invested in transplantation. Nearly all participants finally concluded that although penis transplantation would be worth exploring as a future treatment modality for transgender men, it would be better to optimize present care modalities.

T4: “There are even better alternatives than this ‘extreme’ transplantation. And it is for so far in the future. It is better to focus on other things.”

Healthcare providers doubted if they would do well for (all) their patients by offering this care for this group under the present circumstances.

H5: “Yes, but what I do think is that as physicians you do not have to provide everything there is. […] I am in favor of new things, as long as it does no harm […]. But if it limits us in other medical procedures, we will do harm despite our good intentions.”

Many also doubted whether it was the best choice to invest a possibly large sum of money in this specific treatment. The cisgender men proposed that by investing this money in societal awareness or mental health services the transgender community, as a whole, would benefit more. Some even questioned if such money would be better spent in other areas of reconstructive medicine.

C2: “I think it [the option of penis transplantation, authors] should definitely be explored, but transgender care should be much more on the mental health, rather than the cosmetic and appearance […]. It is surely a much broader topic than just the penis. […] So if you have the money, invest this in societal acceptance. I think a transgender person will benefit more from that than from a real penis.”

C1: “If it [penis transplantation, authors] will come at the expense of plastic surgery for someone with severe burns, I would say ‘do something more important’ [treating the burns instead of performing a penile transplantation, authors].”

## Discussion

In this qualitative study, the ethical considerations and opinions regarding penile transplantation surgery for transgender men have been investigated among different stakeholders. This study is unique as it was to our knowledge the first to yield empirical ethical considerations across the field.

Our findings are largely in line with ethical studies on other nonlifesaving VCAs. The focus groups displayed the many ethical considerations participants had pertaining to this topic. However, there appeared to be only a few underlying principles motivating these considerations. The underlying principles in favor of penis transplantation pertained to the importance of developing personal identity, facilitating autonomy and bodily integrity and postoperative benefits**.** Themes such as improvement of personal well-being, wholeness, gender affirmation, and self-determination were underpinned by these principles.

Supporting the feeling of being whole (or complete) as a person, both mentally and physically, is an important value behind much of gender-affirming care.^32^ Following this principle, individuals have the right to experience their body as whole and in congruence with their identity, and the healthcare system should accommodate to that. Earlier scholars have emphasized the special importance of the penis in experience wholeness as a man.^5^ The penis is a clearly visible body part and has a sexual function and is therefore considered specifically important in supporting personal identity. The importance of the penis in developing one’s personal identity is being used as substantiation in favor of penis transplantation, as the alternatives are thought to insufficiently accommodate to this. A qualitative study by Van der Merwe et al. described the real-life experiences of two South-African penile transplant recipients.^33^ Also in this study, the penile transplants contributed extensively to the positive self-image of the recipients. However, in these particular cases both men lost their penis as a consequence of failed ritual circumcision and suffered from “social death,” in contrast to transgender men who have not had the experience of having been born with male genitalia.

Closely related arguments favoring transplantation are the values relating to autonomy and bodily integrity (eg, self-determinacy) of the recipient. Autonomy is considered among the most important medical ethical principles in healthcare.^29^ Individuals have the right to decide over their own body and healthcare professionals ought to inquire the wishes of the patient and protect their autonomy.

Informed consent is a basic ethical requirement that allows individuals autonomy in making decisions.^29^ Ethically it requires more than conveying technical information: persons also need to consider whether treatments are consistent with their own values.^34^ Providing well-informed consent is recognized to be difficult for novel innovative treatments due to uncertain outcomes, inconsistent reasoning, misunderstanding risks, wishful thinking, or limited imagination.^35^^,^^36^ In case of penis transplantation, a tendency to be (unrealistically) optimistic about innovative procedures may be exacerbated by the emotional distress experienced by transgender men. Such difficulties in decision-making were recognized by the study participants and contributed to their reluctance toward penis transplantation. In addition, the autonomy of a transplant candidate is closely related to the autonomy of others. Receiving such a risky treatment could impact, for example, healthcare affordability and the lives of the recipient’s children and partner. The transgender participants differed in the extent that they would involve such stakeholders into their consideration.

Some participants questioned whether some groups should be able to make an autonomous decision at all, as they were thought not to oversee the consequences, which would however further limit individual autonomy. This is in line with Petit et al (2004), who state that surgeons are the best in making such a decision for patients.^37^ At the same time, other scholars say that a clinician will not always understand the process the patient goes through,^22^ nor may they be able to empathize truly with being transgender in general.^38^ Nonetheless it is believed that a clinician should not always consent to the patient’s wishes.^22^ In some cases the autonomy of the care seeker and provider may be in conflict. Therefore, the right balance between the autonomy of both parties, and the risks and societal costs, must be achieved.

Most frequently mentioned concerns regarding penile transplantation surgery in transgender men, across the participant groups, pertained to the anticipated risks for the person and society at large. Underlying these concerns were some of the main principles of medical ethics: beneficence, nonmaleficence, and justice.^29^

Participants stated that when requiring the transplant recipient to use lifelong immunosuppressive medication one would do harm by predisposing the person to the risk of opportunistic infections of which they might eventually die. This was particularly experienced when the procedure was seen as a non–life-saving intervention. Unlike lifesaving transplantations, the benefits of non–life-savig transplants do not self-evidently outbalance the risks. Caplan and Purves^8^ reported that the challenging ethical issue some VCA transplants raise is whether it is permissible to risk shortening life in order to achieve additional quality of life.

In agreement with some of the opinions of the study participants, Patel et al stated that penile transplantation lacks both life-saving and quality of life–enhancing properties compared to the available alternative, phalloplasty,^39^ and that therefore penile transplantation is not justified as a medical priority, as the potential benefits do not outweigh the risks and costs. Transplantation medicine, however, is a developing field, and others have reported that advances in immunosuppressive medication likely contribute to (changes in) the assessment of risks of complications.^40^ One can state that this development may work in favor of penile transplantation. Follow-up studies of penis transplantation in cisgender men confirm the substantial risks of immunosuppression-related complications.^18^ In addition, participants also feared psychological risks related to rejection or regret***.*** The postoperative course of organ transplants can be characterized by episodes of acute rejection. Such (temporary) physical or physiological rejection can compromise psychological acceptance of the organ.^41^ Although penile transplantation may reduce psychologic burden, the possibility of posttransplant distress is thought to be a substantial risk.^13^^,^^14^^,^^42^ It has been observed that transplant recipients commonly react to their transplanted organ with some degree of estrangement.^41^ In case of hand and face transplants, personal qualities from the donor may be attributed to transplanted organs, leading to this sense of estrangement.^24^^,^^43^ In case of life-saving transplantations, recipients have no choice but to live with the transplanted organ, but with transplantation of nonvital organs, removal is an option^24^ and has been an observed outcome of penis transplantation,^8^^,^^13^^,^^14^ raising some question regarding who could be considered appropriate candidates.^44^ It seems therefore pivotal that recipients will receive substantial (postoperative) counseling to support the process of transplant acceptance.

On the matter of costs, Moodley et al.^45^ noted that state government funding for penile transplantation after botched male circumcision would be very hard to ethically justify in the light of other health priorities. According to these authors it seems more reasonable to prioritize funding of care with larger net effects on the health of the population at large. In line with the aforementioned concerns, our participants debated whether society should pay for penile transplantation in transgender care, since a safer and less costly alternative (phalloplasty) is available.

Another important argument against penile transplantation brought up in all focus groups was the possible negative societal impact penis transplantation for transgender men may have on the willingness to become a donor. This fear related to both the risk of a decline in donor willingness in general as well as availability of donor penises. It has been reasoned that if made aware of the possibility of hand and face donation, registered donors might consider revoking their donor status.^46^ Zhang et al. reported that for families grieving the death of their loved one, discussing the concept of harvesting penile tissue for donation could be distressing.^44^ Obtaining a donor penis due to family reluctance can be experienced as an obstacle during the process of penile transplantation.

As reported in earlier studies, we observed that for all participants, regardless of the participant group, the risks and benefits were weighted differently. These differences in attitudes to individual risks and benefits make some argue that it is best to assess risk/benefit ratios on a case by case basis,^40^ rather than to define general principles**.** In a review on facial transplantation the authors stated that the key to the success of introducing transplant therapy is in the selection of the appropriate patient. Some of the proposed characteristics included being psychologically stable, well-motivated and therapy compliant,^47^ while others required intensive psychologic and psychiatric treatment involving the family members for improving outcome.^48^ Ngaage et al.^26^ state that allocation of life-enhancing grafts such as penile transplants ought to follow that of lifesaving transplantation, with allocation of resources based on equity, priority, and net benefit.^49^^,^^50^

Scholars mention that weighing of risks and benefits and the anticipated social impact are among the most important ethical topic to consider.^23^^,^^25^ Transplant-related complications and immunosuppressive medications are brought up as the most relevant risks.^47^ On the quality of life–improving yet possibly life-shortening aspect of interventions in the field of medicine it was stated: “the art’s most delicate aspect is not to shorten life further, and not to diminish it”.^51^ In some cases of facial transplantation, the (psychological) “handicap” considered by some to be so disabling that the benefits outweigh the risks of the procedure and lifelong treatment.^37^ Other non–life-saving VCAs have also been justified primarily on the basis of quality of life improvements (eg, uterus transplants).^52^ Yet scholars have stated that the consideration between the risks and benefits should be analyzed for each VCA specifically.^25^ In this sense, the degree to which medical professionals and the general public are able to emphasize with the degree of suffering seems to have a major impact on support for VCAs. The ability to emphasize with the distress transgender men experience when having incongruent or insufficient male genitals differs greatly from person to person. Regarding the risk of limited donor willingness, earlier VCA research showed that people are less willing both to donate and to receive a hand, lower extremity, abdominal wall, or a face than solid organs.^53^ Ninety percent of individuals are willing to donate a kidney, whereas 45% would donate a hand/upper extremity and only 39% their face.^54^

After all, the predominant finding was that participants were overall reluctant toward introducing this procedure, with its current characteristics, as part of transgender healthcare. Principles were mostly person-specific rather than stakeholder group–specific. The considerations were mostly based on personal norms and experiences. As a result, some participants were more able to empathize with transgender suffering, with the risks of complications, or with possible societal consequences. Remarkably, within the transgender group the majority of participants expressed their reluctance toward introducing such risky and expensive treatments in order not to lose societal support for transgender care as a whole (in contrast with reducing individual suffering).

## Limitations

A first limitation of the study was the small sample size of the focus groups. This might have resulted in insufficient saturation of some minor considerations. However, since there were three different focus groups of 3 hours, we generally experienced sufficient data saturation, as described above, in the present sample. The described results, extracted from the answers to the questions based on the ethical framework, as described previously, reflect the individual personal opinions of the stakeholders and their ethical view on penile implants. Therefore, some assumptions regarding the impact of penile transplantation might not reflect the current state of post-VCA regimens and associated risks.^55^ Nonetheless, we were able to confirm most of our findings in the literature. Furthermore, the study was set up in a university hospital in X and the present transgender men were not choosing their treatment nor were the cisgender men consciously choosing to be a donor at the time of the study. This could make the results more hypothetical and less generalizable. Another bias may be that the participants were all shortly briefed similarly by the researchers about the new techniques for penile transplantation, which may have directed the participants’ thinking in similar directions. Yet, the information was only presented as a framework, and all participants were free to discuss subjects of their own interest. Also, penile transplantation is not a current option in transgender healthcare and the ethical considerations were therefore largely hypothetical. Last, no feedback sessions were held with the participants, which might have reduced the quality of the interpretations.

## Conclusions

In conclusion, based on the outcomes of different focus groups, the ethical considerations regarding penile transplantation come to terms with psychological well-being, autonomy, the medical risks, the risks for transgender and donor support, and issues regarding equality. An argument in favor was the expected improved personal identity, improved autonomy, and bodily integrity of the recipient as result of aesthetics and functionality. Arguments against penile transplantation were the physical risks related to immunosuppressive medication, psychological burden, and societal impact on the willingness to donate. At this time, penile transplantation was not considered to be of additional value compared to the gold standard phalloplasty, as was broadly supported by the stakeholder groups. However, it remained uncertain how the groups’ considerations and support would change if transplantation medicine were to move toward lower-risk posttransplant immunosuppressive regimens and higher donor availability. Furthermore, ethical considerations such as selection and prioritization of candidates, the donor perspective, and also alternatives such as tissue engineering, should be investigated more extensively. Continuous developments in either field suggest a continuous, more in-depth comparison between current flap reconstruction and tissue engineering along with VCA transplantation options. Therefore, more research regarding ethical concerns is recommended when surgical techniques and immunosuppressive treatments have been further developed.

## Appendix 1

**Focus Group: Medical Specialists**

- Welcome and introduction
- Thematic discussion

*Opening question*

1. What are your first thoughts on implementing penile transplantation in transgender surgery?

*Weighing risks and benefits*

2. Taking into account your experience and expertise in your medical specialty, what would be the greatest benefit of a penis transplant for transgender men?
3. From your own experience, do you foresee disadvantages or possible complications of penile transplantation in transgender men?
4. If someone were technically ready for a penis transplant, who would be particularly entitled to a penis transplant?
5. What social consequences do you foresee after a penile transplantation?

*Personal identity and bodily integrity.*

A penis can play an important role how a trans man experiences his personal identity and feeling complete as a person. A penis is also seen as a body part that has a lot to do with intimacy. A transplanted penis comes from a donor and has therefore belonged to another person. A dilemma could be that the transplanted penis is perceived as foreign by the patient and possibly the partner, as opposed to the body’s own.

6. How would you communicate this to the patient?

*Closing question*

7. Looking back at all that we’ve discussed, do you now have a different view of a penis transplant for transmen? If so, why?

**Focus Group - Transgender Men**

- Welcome and introduction
- Thematic discussion

*Opening question*

1. Would you be interested in a transplanted penis?
2. Do you feel it is an issue within the community?
3. What is your first impression when it comes to transplanting a fully functioning penis as a possibility in transgender surgery?

*Weighing risks and benefits*

4. What would you see as the biggest benefit of the penile transplantation?
5. What would you see as the biggest risk of the penile transplantation?
6. What disadvantage weighs heavily?

*Social importance*

7. How do you think society would react to such a development?
8. To what extent do you have to take society into account?
9. Who would be entitled to a penis transplant?
10. What should preoperative healthcare entail?

*Personal identity and bodily integrity*

11. A transplanted penis is foreign to one’s own body, how would that influence your decision to receive a penis transplant?
12. A penis is a body part which is used for sexual intimacy. What role would a partner play in the decision to receive a transplanted penis?

*Closing question*

13. Looking back at all that we’ve discussed, do you now have a different view of a penis transplant for trans men? If so, why?

**Focus Group: Cisgender Men**

- Welcome and introduction
- Thematic discussion

*Opening question*

1. What are your first thoughts on implementing penile transplantation in transgender surgery?

*Weighing risks and benefits*

2. What would you see as the biggest benefit of the penile transplantation?
3. What would you see as the biggest risk of penile transplantation?

Because it is a transplant from a donor to a recipient, the recipient has to take immunosuppressive medication for the rest of his life to prevent rejection. This medication suppresses the functioning of the immune system, which puts a person at higher risk of other diseases, such as various types of cancer. This can significantly shorten a person’s life expectancy. On the other hand, a penile transplant could improve the quality of a person’s life.

4. If you take into account the life-long immunosuppressive medication, what are your thoughts on penile transplantation?
5. What would be the greatest barrier to undergo penile transplantation yourself?

Think of: rejection, higher chance of other diseases, shortening of life.

*Personal identity and bodily integrity.*

A penis can play an important role in how a transgender man experiences his personal identity and feels complete as a person. A transplanted penis comes from a donor and has therefore belonged to another person.

6. How would you feel about receiving a transplanted penis that belonged to someone else?

A penis is also seen as a body part that has a lot to do with intimacy and contact with others.

7. How would that influence your decision to either receive or donate a penile transplant?
8. What role would a partner play in either situation?

*Donation*

9. What do you think of donating your own penis? Transgender man of biological man disregarded.
10. How would the option to donate a penis affect the donation of other organs in general?
11. Would a penis be any different to donate than a heart or a kidney? If so, why? If so, would that be a problem?
12. If you would donate your penis, would you like to have a phalloplasty (penis constructed of a person’s own tissue) instead?
